# Pharmacogenomics, How to Deal with Different Types of Variants in Next Generation Sequencing Data in the Personalized Medicine Area

**DOI:** 10.3390/jcm10010034

**Published:** 2020-12-24

**Authors:** Alireza Tafazoli, Natalia Wawrusiewicz-Kurylonek, Renata Posmyk, Wojciech Miltyk

**Affiliations:** 1 Department of Analysis and Bioanalysis of Medicines, Faculty of Pharmacy with the Division of Laboratory Medicine, Medical University of Białystok, 15-089 Białystok, Poland; Alireza.Tafazoli@umb.edu.pl; 2 Clinical Research Centre, Medical University of Białystok, 15-276 Bialystok, Poland; 3 Department of Clinical Genetics, Medical University of Białystok, 15-089 Białystok, Poland; natalia.kurylonek@gmail.com (N.W.-K.); rposmyk@gmail.com (R.P.)

**Keywords:** pharmacogenomics, NGS variants, personalized medicine

## Abstract

Pharmacogenomics (PGx) is the knowledge of diverse drug responses and effects in people, based on their genomic profiles. Such information is considered as one of the main directions to reach personalized medicine in future clinical practices. Since the start of applying next generation sequencing (NGS) methods in drug related clinical investigations, many common medicines found their genetic data for the related metabolizing/shipping proteins in the human body. Yet, the employing of technology is accompanied by big obtained data, which most of them have no clear guidelines for consideration in routine treatment decisions for patients. This review article talks about different types of NGS derived PGx variants in clinical studies and try to display the current and newly developed approaches to deal with pharmacogenetic data with/without clear guidelines for considering in clinical settings.

## 1. Introduction: Pharmacogenomics and High Throughput Sequencing Methods

It has been reported for decades that different drugs show different responses and efficacy in diverse individuals or populations. Investigations proved that part of this diversity (20–30%) is because of genetic background and, more precisely, the inheritance of various alleles and variants in genes for drug-metabolizing and transporting (pharmacogenes) or drug target molecules [1]. Pharmacogenetics is the term for the knowledge of diverse drug responses and effects in people, based on their single genes on the genomic profiles. When a group of genes (multiple genes), or whole genome, and other influential genomic events, such as epigenetics will be addressed at once for such investigations, the phrase would be replaced by pharmacogenomics (PGx). Since the starting of employing high throughput sequencing methods, especially next generation sequencing (NGS) technologies, in addition to some comprehensive orthogonal tests, such as genome-wide single nucleotide polymorphism (SNP) arrays in clinical investigations and practice, numerous genetic variants have been introduced in drug-related genes in the human body. Today, close to 100 variants in each people in more than 900 of such genes are mentioned in literature, and the number is increasing continuously [2,3]. There is no doubt that the NGS methods played a significant role in the identification of PGx variants in a clinical research setting and used in the prediction of the response to or adverse effects of drugs, which result in the calculation or estimation of appropriate drug dosage for patients. According to the patient’s responses, the drug outcome could be defined as efficient, inefficient, toxic, and resistant. All of these categories mostly arise from the interaction between the products of many genes in a cellular pathway or between the genes and environmental factors. Hence, genotype-specific therapy could bring huge benefits for drug safety and efficacy in patients in addition to time and cost reduction of treatment approaches for them [4]. The trends led to the practice of personalized therapy and precision medicine implementation in clinical centers. The explosion of examples in the field of pre-emptive and/or patient genotyping shows the true advantages of high throughput sequencing technologies in the PGx area [5,6,7,8]. However, despite the common belief between the physicians and general practitioners in the effects of the genetic landscape on diverse drug responses, if they asked that they order the PGx tests for their patients, less than 15% will answer positively. This is mostly because of the lack of clear guidelines and sufficient clinical evidence for many functional genetic variants (FGVs) in drug-related genes (*FGVs or actionable genetic variants are those alterations in genome, with at least one report for introducing the effects on drug safety and/or efficacy in people. Moreover, the variants found in the research area with strong potential effects on drugs could be considered as FGVs during prescription. However, the latter needs clinical evidence to be influential on treatment decisions by physicians*). Furthermore, the poor knowledge and background of PGx and the different related alleles and variants for many healthcare professionals may directly affect their desire to order the tests.

Yet, several rare and uncommon FGVs can be detected through the PGx tests in both clinical and research areas, especially when comprehensive and high capacity methods, such as NGS, have been utilized [9]. Moreover, it is necessary to distinguish the definition of FGVs and/or uncharacterized variants, such as variants with unknown clinical significance in two distinct genomic medicine areas, PGx, and medical genetics. Although the two concepts are usually mixed and many PGx variants are covered in the medical genetics zone, the first one mostly emphasizes those variants with an impact on pharmacological treatments, while the second group of variants is considered the genetic variations with pathogenicity effects in the human body. For a PGx variant, it might show an interaction with drug dosage modifications or not, but the functional and clinical consequences of a genetic variant may be unknown (does it have pathological consequences?) or well known (it has or not pathological consequences). However, both types of variants will be addressed as the same in NGS primary data analysis steps. To deal with the different genetic variants in PGx profiling of individuals, this review article reviews various NGS derived biomarkers and the possible approaches to use or consider them during the medicine prescription. Those PGx variants with no clear guidelines will be focused on more.

## 2. Different Types of Variants and Their Classifications in Clinical Pharmacogenomics

Both common and rare alleles are demonstrated as the functional biomarkers in PGx clinical practice. Low frequency and rare variants have been shown by 1–5% and lower than 1% minor allele frequency (MAF), respectively, in populations. Moreover, they proved to be very population-specific and the causative elements for diverse drug responses in alternative ethnic groups [10,11]. NGS methods revolutionized the detection of any type of variants in different aspects of genome analysis and profiling, as well as pharmacogenetics and genomic studies. Such investigations reported that most of the FGVs in the clinical PGx setting are Single Nucleotide Variations (SNVs). However, structural variants (SVs), such as Copy Number Variation (CNVs), small Insertion–Deletions (InDels), tandem-substitutions, and the deletion of entire exons are also identified as effective variants in drug responses [12,13]. In addition to wild-type alleles, the functional outcome for each of these variants may cause the individuals to fall into four main groups of responders including poor, intermediate, extensive, and ultra-rapid metabolizers.

Currently, core web-based resources for clinical PGx annotations include Pharmacogenomics Knowledge Base (PharmGKB), the Clinical Pharmacogenetics Implementation Consortium (CPIC), the Pharmacogenomics Research Network (PGRN), and Dutch Pharmacogenetics Working Group (DPWG). These are considered as reference databases that provide information about how human genetic variations affect response to medications. All of the confirmed data about clinically actionable gene-drug associations and genotype-phenotype relationships are sorted properly and available as a guide for personalized medicine implementation by healthcare professionals. However, other modules, such as PharmVar, FINDbase, SuperCYP, SEAPharm, etc. could also be applied when a specific type of gene or drug was on the desk. Nevertheless, according to PGx reference organizations (PharmGKB, CPIC-PGRN, and DPWG), all the diagnosed alleles and variants in a gene-drug interaction, based on the number of published studies and clinical evidence, will be classified in various types of level with clear explanations for each of them (Table 1). However, CPIC has also introduced a new categorization system for PGx level in more detail (Table 2). Generally, different levels of clinical relevance for PGx variants and/or gene-drug pairs will be assigned by the reference entities. All of them have their processes to assign the levels and prioritize approaches for providing the related guidelines. Meanwhile, some recommendations are related to each other (CPIC and PharmGKB) and the others go through it independently (DPWG). For example, the clinical pharmacogenetics implementation consortium (CPIC) allocates the levels for a variant in a gene-drug pair, based on three major criteria from PharmGKB clinical annotation levels of evidence and PGx level for Food and Drug Administration (FDA)-approved drug labels and also if it is nominated to CPIC for consideration. Only those gene/drug pairs that have been the subject of guidelines have had sufficient in-depth review of evidence to provide definitive CPIC level assignments. CPIC also use other considerations for assignment of CPIC level through some essential questions, containing the information of prescribing actionability, the severity of the clinical consequences for ignoring the genetic tests, already subjected gene to other CPIC guidelines, availability of genetic test for the gene, high-risk genetic variants, etc. [14,15]. PharmGKB also creates genotype-based summaries describing the phenotypic impact of the variant and provides the PGx levels from 1A to 4 in combination with four instructive labels as “Testing required”, “Testing recommended”, “Actionable PGx”, and “Informative PGx” via literature reviews while considering population size and statistical significance. The labels state different considerations for the drugs, based on gene/protein/chromosomal variants or phenotypes, and conclude the necessity of pre-emptive genetic testing for genotype/phenotype correlation assays and showing the potential changes in efficacy, dosage, metabolism, or toxicity [16,17]. Finally, the Dutch Pharmacogenetics working group (DPWG) uses the drug-gene interaction outcomes to providing the clinical relevance levels, where the AA is the lowest impact and F is the highest one. The impacts are categorized, based on adverse drug events, decreased therapeutic response, and other clinical effects, result in the allocation of specific scores from 1–7 derived from national cancer institute (NCI) common toxicity criteria and 0–4 level of evidence of gene–drug interaction in the literature [18].

Regarding the abovementioned level of classification for the identified variants, the utilization of NGS platforms for clinical PGx tests brings various types of alleles, which after confirmation and validation processes could be categorized as functional/potential effective variants, fall into “five groups of (1) annotated variants with the clear guideline (i.e., rs1057910 in *CYP2C9* and rs9923231 in *VKORC1* genes for Warfarin). (2) Annotated variants with no clinical guideline (i.e., rs6166 in *FSHR* gene for urofollitropin). (3) Variants with annotation or guidelines for other drugs (i.e., rs9322335 in *ESR1* gene for letrozole while the gene is studying and considered as the estrogen receptor and target molecule for Clomifene). (4) Non-pharmacogenetically annotated variants (i.e., different clinical related variants in *AR* gene as an important target molecule for infertility drugs). And (5) Variants of unknown significance (VUS). The next part will focus on different approaches for such variant interpretation and curation in clinical practice.

## 3. Approaches to Dealing with Diverse Pharmacogenomics Variants

**Figure 1:**
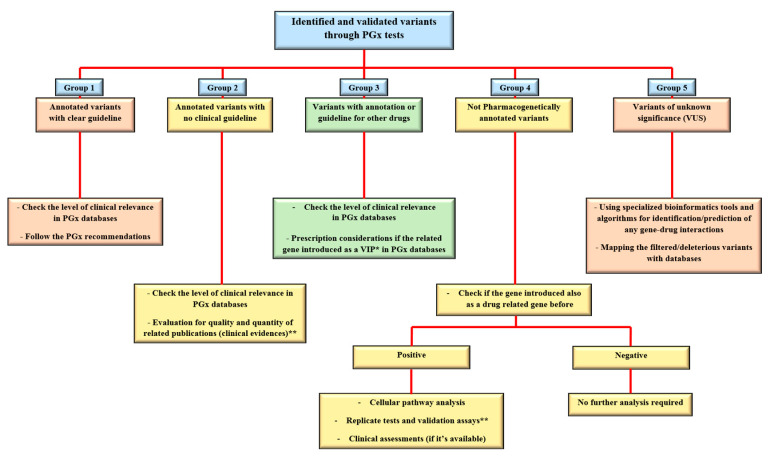
Approaches to deal with different types of PGx variants in clinical centers. After the identification and doing the confirmation tests on a PGx related variant, it could be categorized in one of the main five groups of annotated with PGx guideline, annotated without a guideline, informative for other drugs, not PGx annotated, or variants of unknown clinical significance (VUS). For the annotated variants, checking the level of clinical relevance (Table 1 of the current paper) is the first task to do. Bioinformatics tools are also supporting the analysis of not only VUS but also other types of variants in each group. *Examples for groups 1–5 with explanations are provided in the main text.* * VIP: very important pharmacogene. ** see the text for more details.

To finding any clinical relevance for different groups of PGx variants from the sequencing platforms, standard algorithms, and procedures are introduced by the reference sources (Figure 1). These are the recommendations that indicate the approaches for decoding or predicting the variant functions and the related phenotypes as the diverse drug responses in individuals [22]. From the previous section, group 1 is considered as straightforward, actionable variants in gene-drug pairs with direct prescription recommendations for applying in routine clinical practice. Group 2 are the alleles, consisting of the most common types of identified variants during diagnostic procedures for PGx tests. As the PharmGKB included 19,028 variant annotations, most of the identified markers will fall into this group. Here, the number of clinical evidence in addition to statistical signification (i.e., number of patients in cohort studies) and types of the publications, if they are strong genome-wide association study, well designed replicated report, case report, non-significant study, or only an in-vitro study, would be the important factors for clinical consideration and decisions [23]. The other common scenario for the sequencing results of a pharmacogenetic screening test could be found in group 3, which are variants with the recommendations but not for the researchers/clinicians targeted drugs. Generally, if the related gene is introduced as a very important pharmacogene (VIP) in PGx databases, it is mostly well documented so the related cellular pathways must be analyzed thoroughly. Then the caution and consideration before dosage adjustment are suggested for more accurate implementation of personalized medicine in the clinic [24]. If there is a lack of such documents, more confirmation and validation assessments are necessary before any concerns for the patient’s prescription. Replicate tests in target drugs in such situations consist of various approaches, from looking for the same result in same/different ethnic groups to implementation of laboratory confirmation tests. However, alternative approaches have also been introduced for PGx findings validation, if replication studies for gene-drug interactions proved to be difficult and costly for some cases [25]. In the end, consulting with gene experts or experienced clinical pharmacologist in the gene–drug interaction field is necessary. So far, reference databases have explained the approaches to deal with variants in group one to three. However, many genetic variations may be classified in group 4, which is introduced as disease-associated biomarkers and placed into the different genomic databases, such as ClinVar, dbGaP, HapMap, gnomAD, COSMIC, etc. (as causative or pathogenic variants), but there is no PGx report for them. This is mostly happening during more comprehensive genomic profiling of individuals for decoding any PGx markers. In such a situation, the first step could be the evaluation of the gene, if it is introduced as drug related in literature and databases before. The positive result may follow the approaches for group 3 as well. If there is any, also clinical assays would help provide evidence in both groups 2 and 3 of variants during the clinical decision making.

The last types of variants (group 5) are the novel and unreported variations in databases (ClinVar, HGMD, PharmGKB), but found in a PGx test mostly through comprehensive methods, such as whole exome or whole genome sequencing (WES and WGS), with no clue for their function in causing a particular phenotype. Moreover, incidental findings (IFs) are the group of known variations, but not related to specifically investigated phenotype, and accidentally revealed during a sequencing test. Both the VUS (novel variants) and IFs will be manageable with higher accuracy by the combined usage of highly specialized bioinformatics pipelines to find any possible interaction with drug responses in patients. IFs are mostly displayed as the annotated functional drug-related variants in pharmacogenes and potentially useful markers if the appropriate genomic analysis and accurate genotype–phenotype correlations are performed subsequently [26]. We will address this topic in detail in the following section.

## 4. Approaches to Dealing with Novel Pharmacogenomics Variants

As the majority of revealed variants through implementation of broad range high throughput sequencing tests could be categorized in group 4 and 5 (the most challenging groups), the process of identifying clinically relevant PGx variants from complex genomic data mostly concerns about the detection of any potential FGVs in these two categories. The procedures usually start with digging the variant call format (VCF) file for filtration of variants and selection of those alterations, which come from drug-related genes. Based on the employed sequencer machine and the selected platform for PGx data clinical assessment, different types of variants are available in subsequent result analysis (SNVs and/or CNVs from coding and noncoding/regulatory parts of the genome). Routine silico analysis is considered for filtration of NGS derived pharmacovariants data at the first step (including the quality assessments, segregation studies, zygosity mapping, and allele phasing, etc.). Next, the selected variants go for pathogenicity and functional annotation analysis through the utilization of prediction algorithms in both common (i.e., *SIFT*, *PolyPhen2*, *MutationTaster*) and PGx dedicated tools (i.e., *Stargazer*, *Aldy*, *Astrolabe*) [27,28,29]. As the final stage, computational and in-vitro confirmation studies can aid in the identification of prediction’s sensitivity, specificity, and accuracy level. This is usually implemented via performing the homology modeling, Sanger sequencing, and cell culture modifications. The other approach is the replicate study in an independent validation cohort.

Examples for the generation of clinical recommendations for the variants using in silico analysis of WGS PGx data were done before. The related studies showed the PGx dosage recommendations are heavily influenced by the higher availability of genotyping results, which may lead to more clinical evidence too [30]. Yet, the most important barrier to routine implementation of NGS technologies for PGx tests in clinical centers is the huge amount of uncertain and unknown significant variants in the results (group 5), which need to be confirmed and validated before considered as the influential elements in treatment decision and prescription modification. In addition to some basic problems in using NGS methods, such as poor coverage of the specific parts of the genome, false-positive results in short reads, ignoring many non-coding variants in targeted panels and WES, missing some homopolymer regions, pseudogenes, and GC rich, diverse efficiency for genome capturing due to the utilization of different kits and reagents, etc. [31], any novel or incidental markers still must go through the different validation steps, to be connected to drug-related phenotypes in patients. While looking for previous clinical reports and similar investigations, current approaches in dealing with PGx variants in group 5 are including the computational methods and in-vitro functional analysis of the variants. As the number of altered alleles could be high in NGS data, applying the computational analysis techniques and starting with categorizing, filtering, and functional annotating the variants across the RefSeq and other databases, such as dbSNP or dbNSFP, by special bioinformatics tools, such as *VAT*, *VarAFT*, *ANNOVAR*, etc., is inevitable. Then, the prediction of potentially damaging, deleterious, and/or functionally neutral non-synonymous variants will be performed via the algorithms as mentioned earlier. Currently, the mutual beliefs for PGx data analysis are the combined utilization of 6 to 7 of such prediction tools and choosing those variants, which are commonly introduced as pathogen/likely pathogen in all applied software, according to reliable reference guidelines, such as those given by ACMG, CAP, and CPIC [27]. While there is no universal and widely accepted functional prediction software package, the number of introduced PGx specific analysis tools, such as *Stargazer*, *Astrolabe, PharmCAT*, *PHARMIP*, etc., are increasing rapidly in a fast-developing mode. Hence, integrating them in applied algorithms seems necessary. Table 3 listed some of these special data mining and visualization tools, which are used or considered to be useful in PGx data management. We will talk about the limitations of common analysis facilities later in the discussion section. Next is the pathway mapping of the selected variants against the general and specialized free reference sources, such as PharmGKB, String-db, DAVID, KEGG, etc., to find out about the potential gene–drug and protein–protein interactions. Finally, allele frequency and population derived variant analysis could be achieved through comparing with comprehensive surveys (1000 genome, ExAc, HapMap, ESP, gnomAD, GME) [32]. Moreover, laboratory confirmative assays and characterization could be implemented for just top prioritized functional variants, to roll out any false-positive result and be assured of the real harmful effects on drug response. The final clinical assessments (if it’s available) support the necessity for genotype–phenotype correlation procedures too.

## 5. Discussion

NGS technologies have been used in several PGx studies in recent years. Based on the employed platforms, the acquired data analyzed through different approaches. Due to the lower amount of identified variants (mostly known alleles), finding the FGVs and phenotype prediction is usually easier when targeted sequencing for a specific set of the gene (panels) is performed as the selected method. WES and WGS, however, show a lot of obstacles when applied for a PGx analysis and this is mainly because of the huge number of functionally unknown and unreported alterations in a patient’s genetic profile [70]. Moreover, some intrinsic and substantial complications for PGx tests including the presence of germline mutations with necessary haplotype detection and phase definition in patients, going through specific pharmacogenes with a role in different sophisticated cellular pathways (i.e., *ACE*), following environmental and epigenetic modifications on drug-related genes, working with challenging and problematic variants, in particular drug-related genes (i.e., *CYP2D6* with close pseudogenes and many unknown and novel variants in diverse populations, different functional tandem repeat variants in the non-coding part of *UGT1A1* gene, etc.), and most important of them the lack of previous knowledge on possible phenotype modifications for many genetic changes (as PGx is a pre-emptive genotyping test in numerous cases) can potentially increase the difficulties in variant analysis and pose the clear effects on changing the drug responses in individuals. Albeit, providing more genotype to phenotype translation methods by reference organizations and guideline developers will result in more consistent genotype interpretation in both clinical and research area [71].

Despite the challenges, the number of publications for NGS derived PGx data analysis are still significant. Gordon et al. successfully identified common, rare, and novel variants in 84 clinically actionable drug-related genes in more than 280 individuals through a targeted resequencing custom panel. They used deep coverage of the known genes to follow both previously recognized and possible novel variants. New potentially deleterious nonsense and missense variants across some VIPs were selected for more genotype-phenotype association studies to find any relation with particular traits (group1, 2, and 5 of the PGx variants). Moreover, actionable plus rare unreported variants in absorption, distribution, metabolism, and excretion (ADME) core genes revealed in 114 drug genes in 376 people by Han and colleagues. The number of variants in each gene (normalized based on gene length), MAF, and novelty assessed and compared to open genotyping datasets (group2, 4, and 5). In silico functional assessments performed by the prediction tools, such as *SIFT*, *PolyPhen2*, and *CAAD,* and deleterious rare-novel variants in some of VIPs evaluated by in-vitro analysis to find impaired functions evidence. Moreover, additional and novel faraway variants (group 5), contributed to the alteration of estrogen receptor binding site and breast cancer risk identified in 400 patients by NGS deep sequencing and functional genomics. As the number of investigated genes was low, any novel PGx variant was confirmed through the laboratory tests, such as chromatin immunoprecipitation (ChIP), gene expression analysis, and protein degradation assays [72,73,74]. Other utilizations also brought more unprecedented results for clinical PGx investigations. For example variants and haplotype detection of challenging ADME genes were successfully achieved in three core pharmacogenes (*CYP2D6*, *HLA-A,* and *HLA-B*) by applying the long read sequencers (group1 and 2). All the SNVs, CNVs, and InDels were revealed through the utilization of customized long-range PCR and the subsequent NGS machine (MinION nanopore sequencer) [75]. Moreover, 17,733 ADME variants per individual were detected in 231 genes. In addition to known PGx markers, the latter included 1012 novel variants with potential deleterious functions identified in exons, introns, gene promoters, and proximal regulatory regions. The authors reanalyzed WGS provided data to find different PGx markers in close to 500 individuals. In silico analysis used the ANNOVAR tool for annotation and dbSNP137 and Complete Genomics public server for novelty assessments. Functional assays were also predicted via SIFT and Provean algorithms (group1, 2, 4, and 5) [12]. In another effort, whole genome sequencing (WGS) in PGx analysis revealed 227 common and 466 rare population-specific potentially functional SNVs, including 74 novel variants in 437 drug genes (group1, 2, and 5). Variant analysis computational workflow consisted of ANNOVAR and dbSNP138 for variant annotation, *SIFT,* and *PolyPhen2* for functional effect analysis of novel non-synonymous coding SNVs, mapping the deleterious variants with PharmGKB and DrugBank, and finally *PLINK* and *VCFTools* for reaching allele frequencies and validation through 1000 genome and HapMap databases. In the end, a drug pathway map for functionally impaired pharmacogenes displayed, using identified deleterious variants [32]. Even the PGx-specific panel with high accuracy designed and identified clinically relevant variants in 39 genes including *CYP2D6* CNV and UGT1A1*28 TAA repeats in promoter in addition to allele frequency and homozygosity in 235 patients. Common in-vitro and bioinformatics tools used for both known and novel variant detection rate accuracy and sensitivity (group1, 2, and 5) [76]. Finally, a comprehensive usage for NGS methods can be found in Price and his team effort, which applied exome sequencing for 21,000 human genes and revealed novel genetic loci with a strong association with on-treatment reactivity and hereditability of platelet and clopidogrel response. Once again, novel loci and related variants in addition to known PGx markers were depicted by common data interpretation pipeline and proved the NGS methods as a powerful approach in unavailing PGx variants in clinical studies [77].

Two important points could be mentioned from the above investigations as well. As the majority of functional prediction tools and algorithms are relying on evolutionary conservation and therefore will not be completely fit with the pharmacogenes (poorly conserved) and show low predictive accuracy as the conventional algorithms (up to 50%), most of the studies emphasize combined utilization of such tools in in silico phenotype prediction for novel variants and introduced various software in each report. This may remind the necessity of the attitude for new PGx data in high throughput sequencing methods, as they are not observable in many cases (pre-emptive genotyping). Recent efforts, however, have been focused on developing new pharmacogene optimized frameworks with more relation to PGx data assessment through the integration of specific algorithms or presenting the allele dedicated for pharmacovariant calling and showed to be more compatible with ADME genes with a higher rate of sensitivity and specificity (90–99%) [27,63]. Other PGx specialized projects are also recently developed a pharmacogenomics clinical annotation tool (*PharmCAT*) and tried to reveal which patients in a clinical dataset include the variants of interest [65].

The second point is the ability of NGS technologies to the detection of any kinds of PGx variants in clinical practice. They have introduced several novel PGx markers successfully and the fact may indicate the faster incorporation of PGx test results into the future precision medicine as well. However, there are still essential issues with high importance in the field, which need to be addressed properly. For example, if the particular novel variant causes a loss of function or gain of function effects on the related protein(s) (making a poor or rapid metabolizer) in tested individuals and also possible misinterpreting of VUS in the result, which may lead to ignore or miss the functional variants in pharmacogenes. Such complexities must be followed by the in-vitro assessments in addition to appropriate pre and post-test counseling for individuals [28,78].

The intricacies are not limited to the detection of variants, but the nature of drug actions according to particular alleles too. Investigations displayed the dual or multiple impacts of some specific pharmacovariants toward the different diseases and/or drugs (Table 4). Furthermore, a certain drug could be the substrate for more than one P450 family and metabolized by different enzymes (i.e., CYP1A2, CYP2C19, and CYP2D6 for antidepressant amitriptyline) [79]. Such scenarios complicate the true functional assessment of pharmacovariants, especially in high throughput sequencing data. Because of that, a comprehensive literature search, replicate studies, and wet lab analysis of the newly identified genetic markers in drug-related genes must be taken into account before any prescription considerations in the clinical setting.

## 6. Conclusions

The field of pharmacogenomics faces several challenges throughout the process of the identification of pharmacogenomic variants and their implementation in clinical practices. Many of these challenges arise at the genomics level, including the statistical considerations associated with the design of the clinical trial and genome-wide association studies (GWAS), a large number of candidate variants compared to available samples (p > n), the lack of reproducibility in independent studies and determining the functional impact of variants on drug response. In the age of PGx and personalized drug therapy, using the high throughput sequencing approaches will assist the translation of different pharmacovariants into clinical care. As mentioned before, for moving genomic medicine toward personalized drug therapy, there should be a genetic screening test, which fits all ethnicities [12]. NGS, as a time and cost-effective and highly accurate genotyping method, shows the huge benefits for patients PGx clinical assessments. Hence, it would be highly possible for the investigators and clinicians to encounter new and rare population-specific variants during a PGx test. To deal with different NGS derived PGx variants in clinics, all healthcare professionals need to know the classification and interpretation algorithms for such markers properly.

## Figures and Tables

**Table 1:** Different levels of clinical relevance for pharmacogenomics (PGx) variants in reference organizations.

*Reference Organization*	*PGx Level*	*Summary of Description*	*Reference*
**PharmGKB**	***1A***	Variants in this level are annotated and have a clear and endorsed guideline while showing a strong role in gene-drug interactions.	[19]
	***1B***	Annotated variant with strong evidence in the literature. Gene-drug association shows strong effects.	
	***2A***	The annotated variant is in a VIP *, so functional significance is more likely.	
	***2B***	Annotated variant but in moderate evidence of an association. There is no reliable replicated study in form of statistical significance or well-designed in size.	
	***3***	Annotated variant in a single study or multiple studies with no similar associations between the variant and the drug.	
	***4***	Annotated variant but in a case report and non-significant study or just in an in-vitro assay.	
**CPIC**	***A***	Variants in this level oblige a change in related drug prescription. Strong clinical evidence and genotype-phenotype correlations exist.	[20]
	***B***	Evidence is weak for the variant but still genotyping may be useful for alternative prescribing.	
	***C***	Different levels of evidence are mentioned in various publications for the variant. No prescribing actions are recommended. Mostly suitable for genes that are commonly included in clinical or DTC ** tests.	
	***D***	Weak evidence and conflicting data are introduced for the variant. Clinical actionability is unclear. No prescribing actions are recommended.	
**DPWG**	***AA***	Variants with no significant clinical or kinetic effects.	[21]
	***A***	Variants with minor clinical effects and kinetic effects.	
	***B***	Variants with mild clinical effects.	
	***C***	Variants with moderate clinical effects.	
	***D***	Variants with stronger clinical effects than level C.	
	***E***	Variants with severe clinical effects as the failure of lifesaving therapy or life-threatening complications.	
	***F***	Variants with most severe clinical effects, death is anticipated.	
	***		
	***4***	There are good quality published studies for the variant/gene.	
	***3***	There are moderate quality published studies for the variant/gene.	
	***2***	Well documented case reports exist for the variant/gene.	
	***1***	Published incomplete case reports for the variant/gene.	
	***0***	Data on file.	
	***???***	No evidence.	

* VIP: very important pharmacogene, ** DTC: direct to consumer, *** Separate the two different levels definitions of the DPWG.

**Table 2:** Clinical Pharmacogenetics Implementation Consortium (CPIC) new level of clinical relevance for gene/drug interactions.

*Cpic Level*	*Clinical Context*	*Level of Evidence*	*Strength of Recommendation*
**A**	Genetic information should be used to change the prescribing of the affected drug.	The preponderance of the evidence is high or moderate in favor of changing prescribing.	At least one moderate or strong action (change in prescribing) is recommended.
**A/B**	Preliminary review indicates it is likely that the definitive CPIC level will be either A or B.	Full evidence review is needed to assess the level of evidence, but prescribing actionability is likely.	Full review by expert guideline group to assign strength of recommendation.
**B**	Genetic information could be used to change prescribing of the affected drug because alternative therapies/dosing are extremely likely to be as effective and as safe as non-genetically based dosing.	The preponderance of the evidence is weak with little conflicting data.	At least one optional action (change in prescribing) is recommended.
**B/C**	Preliminary review indicates it is likely that the definitive CPIC level will be either B or C.	Prescribing actionability based on genetics is not clear without further evidence review.	Full review by expert guideline group to assess the strength of recommendation.
**C**	There are published studies at varying levels of evidence, some with mechanistic rationale, but no prescribing actions are recommended because (a) dosing based on genetics makes no convincing difference; (b) alternatives are unclear, possibly less effective, more toxic, or otherwise impractical; or (c) few published studies or mostly weak evidence and clinical actions are unclear. Most important for genes that are subject to other CPIC guidelines or genes that are commonly included in clinical or DTC tests.	Evidence levels can vary.	No prescribing actions are recommended.
**C/D**	Preliminary review indicates it is likely that the definitive CPIC level will be either C or D.	Evidence levels can vary.	No prescribing actions are recommended.
**D**	There are few published studies, clinical actions are unclear, little mechanistic basis, mostly weak evidence, or substantial conflicting data. If the genes are not widely tested clinically, evaluations are not needed. Criteria for “widely tested” includes: 1) College of American Pathologists (CAP) proficiency testing is available; 2) gene is in disease-specific panels (e.g., pain, psychiatric, cancer, etc.); or 3) evidence exists for implementation of the gene into clinical practice (CPIC member feedback, publications, etc.).	Evidence levels can vary.	No prescribing actions are recommended.

Adopted from cpicpgx.org/.

**Table 3:** Special data mining and visualization tools and algorithms, used in PGx data analyzing and phenotype prediction.

*Software*	*Applications*	*Link*	*Reference*
SIFT	SIFT (Sorting Intolerant From Tolerant) is an online program that predicts whether an amino acid substitution affects protein function based on sequence homology and the physical properties of amino acids.	https://sift.bii.a-star.edu.sg/	[33]
PolyPhen-2	PolyPhen-2 (Polymorphism Phenotyping v2) is a tool that predicts the possible impact of an amino acid substitution on the structure and function of a human protein using straightforward physical and comparative considerations.	http://genetics.bwh.harvard.edu/pph2/	[34]
LOFTEE	Loss-Of-Function Transcript Effect Estimator is a tool to identify LoF (loss-of-function) effects of variations. LOFTEE also makes predictions of another splice (OS) variants that may cause LoF by disrupting normal splicing patterns.	http://www.atgu.mgh.harvard.edu/resources/software/	[35]
VAT	Variant Annotation Tool is a computational framework to functionally annotate variants in personal genomes using a cloud-computing environment.	http://vat.gersteinlab.org/	[36]
VarAFT	Variant Annotation and Filter Tool is for the identification of disease-causing mutations in human genetics. The software improves annotation and filtration steps.	https://varaft.eu/	[37]
EV mutation	An online free tool for predicting the mutation effects from sequences.	https://marks.hms.harvard.edu/evmutation/	[38]
UCSF chimera package	UCSF Chimera is a highly extensible program for interactive visualization and analysis of molecular structures and related data, including density maps, supramolecular assemblies, sequence alignments, docking results, trajectories, and conformational ensembles. High-quality images and animations can be generated. The Resource for Biocomputing, Visualization, and Informatics (RBVI) and its precursor, which is interactive software tools and advanced web-based computational resources that provide integrated visualizations and analyses of molecular structures and related non-structural biological information.	https://www.cgl.ucsf.edu/chimera/	[39]
ICM-Molsoft	ICM-Pro empowers a biologist or chemist by providing a high-quality protein structure analysis, modeling, and docking desktop software environment. Main features include: analyze sequences and alignments, inspect protein structure, study pockets, and bound ligands and drugs, create surfaces, calculate electrostatics, make mutations, predict ligand binding sites, predict protein–protein interaction sites, perform small molecule and protein–protein docking, and design ligands.	http://www.molsoft.com/icm_pro.html	-
EVfold	EVfold uses an evolutionary variation to calculate a set of co-evolved residue pairs in a protein family using a global approach called maximum entropy, formally similar to partial correlations.	http://evfold.org/evfold-web/evfold.do	[40,41]
xBrowse	xBrowse is a platform for studying rare genetic diseases. It was built to provide genetic researchers and clinical geneticists a collaborative way to search for the causes of genetic disease using exome sequencing data. xBrowse accepts as input a set of variant calls from a whole exome or whole genome sequencing study for further processing and annotation. Currently, the only accepted input format is a VCF file produced by the GATK pipeline.	http://www.atgu.mgh.harvard.edu/resources/software/	.
PLINK	PLINK/SEQ is an open-source C/C++ library for working with human genetic variation data. The specific focus is to provide a platform for analytic tool development for variation data from large-scale resequencing and genotyping projects, particularly whole-exome and whole-genome studies. It is independent of (but designed to be complementary to) the existing PLINK package.	https://atgu.mgh.harvard.edu/plinkseq/	[42]
SKAT	SKAT is a Single Nucleotide Polymorphism (SNP)-set (e.g., a gene or a region) level test for association between a set of rare (or common) variants and dichotomous or quantitative phenotypes, SKAT aggregates individual score test statistics of SNPs in a SNP set and efficiently computes SNP-set level *p*-values, e.g., a gene or a region-level *p*-value, while adjusting for covariates, such as principal components to account for population stratification. SKAT also allows for power/sample size calculations for designing sequence association studies.	www.hsph.harvard.edu/skat	[43]
Mutation Assessor	This server predicts the functional impact of amino-acid substitutions in proteins, such as mutations discovered in cancer or missense polymorphisms. The functional impact is assessed based on the evolutionary conservation of the affected amino acid in protein homologs.	http://mutationassessor.org/r3/	[44]
MutationTaster	MutationTaster is a free web-based application to evaluate DNA sequence variants for their disease-causing potential. The software performs a battery of in silico tests to estimate the impact of the variant on the gene product/protein.	http://www.mutationtaster.org/	[45]
PANTHER	The PANTHER (Protein ANalysis THrough Evolutionary Relationships) Classification System was designed to classify proteins (and their genes) to facilitate high-throughput analysis. PANTHER is defined as a method to predict the functional effect of missense variants based on sequence information.	http://www.pantherdb.org/	[46]
PhD-SNP	An SVM-based classifier for the prediction of variant pathogenicity according to sequence profiles.	http://snps.biofold.org/phd-snpg/	[47]
Varscan2	An analysis tool, for the detection of somatic mutations and copy number alterations (CNAs) in exome data from tumor–normal pairs.	http://varscan.sourceforge.net/	[48]
SPLINTER	Detects and quantifies short Insertion–Deletions (InDels) and substitutions in large pools. SPLINTER allows accurate detection and quantification of short insertions, deletions, and substitutions by integrating information from the synthetic DNA library to tune SPLINTER and quantify specificity and sensitivity for every experiment to accurately detect and quantify InDels and substitutions.	https://omictools.com/splinter-tool	[49]
GeneSplicer	GeneSplicer is a new, flexible system for detecting splice sites in the genomic DNA of various eukaryotes and predicting the variant effects on the related protein(s).	http://www.cbcb.umd.edu/software/GeneSplicer/gene_spl.shtml	[50]
NMD Classifier	NMD is a tool for systematic classification of nonsense-mediated decay events for either annotated or de novo assembled transcripts.	https://sourceforge.net/projects/transcriptome-analysis/files/NMD_Classifier.tar.gz	[51]
mrSNP	mrSNP provides a web service for researchers working especially with RNA-Seq Data, to predict the impact of an SNP in a 3UTR on miRNA binding.	https://tools4mirs.org/software/mirna_snp_analysis/mrsnp/	[52]
GenoCanyon	GenoCanyon is a whole-genome functional annotation approach based on unsupervised statistical learning. It integrates genomic conservation measures and biochemical annotation data to predict the functional potential at each nucleotide, both in coding, and non-coding regions.	http://genocanyon.med.yale.edu/	[53]
ANNOVAR	ANNOVAR is an efficient software tool to utilize up-to-date information to functionally annotate genetic variants detected from diverse genomes (including human genome hg18, hg19, hg38, as well as mouse, worm, fly, yeast, and many others). Given a list of variants with chromosome, start position, end position, reference nucleotide, and observed nucleotides, ANNOVAR can perform: - Gene-based annotation; - Region-based annotation; - Filter-based annotation, etc.	http://annovar.openbioinformatics.org/en/latest/	[54]
CADD	CADD is a tool for scoring the deleteriousness of single nucleotide variants as well as insertion/deletion variants in the human genome. It integrates multiple annotations into one metric by contrasting variants that survived natural selection with simulated mutations. C-scores strongly correlate with allelic diversity, the pathogenicity of both coding and non-coding variants.	https://cadd.gs.washington.edu/	[55,56]
Provean	Provean is a software tool that predicts whether an amino acid substitution or InDel has an impact on the biological function of a protein. It is useful for filtering sequence variants to identify non-synonymous or InDel variants that are predicted to be functionally important.	http://provean.jcvi.org/index.php	[57,58]
ESEfinder	ESEfinder is a web-based resource that facilitates rapid analysis of exon sequences to identify putative exonic splicing enhancers, responsive to the human SR proteins SF2/ASF, SC35, SRp40, and SRp55, and to predict whether exonic mutations disrupt such elements.	http://krainer01.cshl.edu/cgi-bin/tools/ESE3/esefinder.cgi?process=home	[59]
VarSeq	VarSeq is an intuitive, integrated software solution for tertiary analysis of next generation sequencing (NGS) data. With VarSeq workflows can be automated and analyzing variants for gene panels, exomes, and whole genomes is possible. Moreover, the tool shows the ability to integrate with new resources and databases for advanced and customized variant analysis.	https://www.goldenhelix.com/products/VarSeq/	[60]
FATHMM	A high-throughput web-server capable of predicting the functional consequences of both coding variants, i.e., non-synonymous single nucleotide variants (nsSNVs), and non-coding variants in the human genome.	http://fathmm.biocompute.org.uk/	[61]
GERP++	Genomic Evolutionary Rate Profiling (GERP) identifies constrained elements in multiple alignments by quantifying substitution deficits.	http://mendel.stanford.edu/SidowLab/downloads/gerp/	[62]
SiPhy	SiPhy implements rigorous statistical tests to detect bases under selection from multiple alignment data. It takes full advantage of deeply sequenced phylogenies to estimate either unlikely substitution patterns as well as slowdowns or accelerations in mutation rates.	http://portals.broadinstitute.org/genome_bio/siphy/index.html	-
Stargazer	Stargazer is a bioinformatics tool for calling star alleles (haplotypes) in PGx genes using data from NGS or SNP array. Stargazer can accept NGS data from both whole genome sequencing (WGS) and targeted sequencing. Stargazer identifies star alleles by detecting SNVs, InDels, and SVs. Stargazer can detect complex SVs including gene deletions, duplications, and hybrids by calculating paralog-specific copy numbers from read depth.	https://stargazer.gs.washington.edu/stargazerweb/	[63]
PharmCAT	A tool to extract all CPIC guideline gene variants from a genetic dataset (represented as a VCF file), interpret the variant alleles and generate a report.	https://github.com/PharmGKB/PharmCAT	[64,65]
PHARMIP	An in silico method to predict genetics that underpin adverse drug reactions. The tool can be used to reveal genetic risk factors for certain drug ADRs.	http://www.lilab-ecust.cn/pharmmapper/	[66]
PharmVar API	An online source for access to all or selected data of the Pharmacogene Variation Consortium (PharmVar) database.	https://www.pharmvar.org/documentation	-
Astrolabe	Astrolabe is software for the translation of whole genome sequence data into pharmacogenetic information that can be used to guide medication selection, dosing, and prescription. It was initially developed under the name Constellation for the *CYP2D6* gene, then extended to *CYP2C9* and *CYP2C19* with additional genes in the process of being validated. Astrolabe is integrated with the PharmVar database	https://childrensmercy.org/genomesoftwareportal/Software/Index/	[67]
Aldy	Aldy performs allelic decomposition of highly polymorphic, multi-copy genes by using whole or targeted genome sequencing data. For a large diverse sequencing data set, Aldy identifies multiple rare and novel alleles for several important pharmacogenes, significantly improving upon the accuracy and utility of current genotyping assays.	http://aldy.csail.mit.edu.	[68]
Cypripi	An algorithm to computationally infer *CYP2D6* genotype at base pair resolution from high throughput sequencing data. It can resolve complex genotypes, including alleles that are the products of duplication, deletion, and fusion events involving *CYP2D6* and its evolutionarily related cousin *CYP2D7*.	http://sfu-compbio.github.io/cypiripi/	[69]

**Table 4:** Examples of different outcomes for one particular allele/diplotype of *CYP2D6* in different disorders and drugs.

*Disease/Disorder*	*Drug*	*Gene*	*Diplotype or Allele*	*Decreased Response*	*Increased Response*	*Low Plasma Concentration*	*High Plasma Concentration*	*Toxicity*	*Level of Evidence*	*Reference*
Depressive Disorder Mental Disorders	Paroxetine	*CYP2D6*	*1/*1xN ^#^	-	✓	✓	-	-	1A	[80]
Nausea and Vomiting after Chemotherapy	Ondansetron	*CYP2D6*	*1/*1xN	✓	-	-	✓	?	1A	[81]
Mental Disorders	Desipramine	*CYP2D6*	*1xN	✓	-	-	✓	?	2A	[82]
Alzheimer Disease	Donepezil	*CYP2D6*	*1/*1xN	-	✓	✓	-	-	3	[83]
Pain	Codeine	*CYP2D6*	*1/*1xN	-	✓	✓	-	✓	1A	[84,85]

# Gene duplication, which resulted in ultra-rapid metabolizer. * is a standardized nomenclature system used for various haplotypes and alleles in Cytochrome P450 family pharmacogenes. The level of evidence is adopted from PharmGKB [19]. *✓: Yes, ?: unknown, -: not applicable.*

## Abbreviations

ACMG	American College of Medical Genetics and Genomics
ADME	Absorption, Distribution, Metabolism, and Excretion
CADD	Combined Annotation-Dependent Depletion
CAP	College of American Pathologists
CNV	Copy Number Variation
CPIC	The Clinical Pharmacogenetics Implementation Consortium
DPWG	Dutch Pharmacogenetics Working Group
FDA	Food and Drug Administration
FGV	Functional Genetic Variation
GWAS	Genome-Wide Association Studies
IF	Incidental Findings
InDel	Insertion–Deletion
MAF	Minor Allele Frequency
NCI	National Cancer Institute
NGS	Next Generation Sequencing
PDG	Pharmacogenomics Dosage Guidelines
PGRN	The Pharmacogenomics Research Network
PGx	Pharmacogenomics
PharmCAT	Pharmacogenomics Clinical Annotation Tool
PharmGKB	Pharmacogenomics Knowledge Base
Provean	Protein Variation Effect Analyzer
SNP	Single Nucleotide Polymorphism
SNV	Single Nucleotide Variation
SV	Structural Variants
VAT	Variant Annotation Tool
VarAFT	Variant Annotation and Filter Tool
VCF	Variant Call Format
VIP	Very Important Pharmacogene
VUS	Variants with Unknown clinical Significance
WES	Whole Exome Sequencing
WGS	Whole Genome Sequencing
